# A Case of Tetanus Affecting the Muscles of Jaws Only

**Published:** 1895-09

**Authors:** N. C. Mitra

**Affiliations:** Ranchi, Bengal, India


### A CASE OF TETANUS AFFECTING THE MUSCLES OF JAWS ONLY.

BY N. C. MITRA, M.A., M.B.,

RANCHI, BENGAL, INDIA.

Tetanus is defined as a disease consisting in an excited state of the spinal cord and the medulla oblongata, in fact of the whole true spinal system, giving rise to painful and continued spasms of the voluntary muscles. The question is, whether the disease does ever confine itself to the muscles of jaws only, leaving all other voluntary muscles in their normal states. Authorities are quite unanimous on the point that tetanus commences as lockjaw, but gradually affects all the other voluntary muscles. A case came under my treatment in which the patient had lockjaw as the result of an arrow wound received twenty-one days previously. There were no other symptoms of tetanus, excepting trismus, or spasms of the masseters. The patient ultimately succumbed to the attack. The case may be detailed as below:

Poonia, male, aged thirty-five, was admitted to the Ranchi Imperial Hospital on the 3d of April, 1894, suffering from an arrow wound of the right thigh.

The bow and arrow were set in a jungle to kill tigers; the patient, while back from the jungle, passed by the way where the arrow was fixed and was struck by it. It may be interesting to the reader of this paper to know how bows are set in Indian jungles for the purpose of killing tigers and other ferocious animals. The arrow-heads are smeared with vegetable poisons. The bow and arrow are set with such precision that the arrow, when shot, will not strike any other animal excepting a tiger. The *shikarees* (or men engaged in this business) take care to find out the paths in the jungle frequented by the tigers and the approximate height of these animals. Having satisfied themselves as to these points, they betake to setting the bow with the arrow fixed. The arrow-head is made to point towards the path trodden by the tiger, and a fine string is attached to the bow with such a mechanism that the least touch on the string will let free the arrow from the bow. The string, moreover, is fixed at such a height from the ground that the arrow when shot will strike the chest of the tiger. If cows or buffaloes happen to stray along the path and perchance come against the string, the arrow passes underneath their abdomen, whereas goats or sheep are not high enough to touch the string. The villagers, of course, are kept fully acquainted with these, and they avoid passing that way. If by chance any go that way the arrow strikes him in the thigh. Death follows from injury to the vital parts, as well as from effects of poison on the arrow-head.

The patient was not aware of the setting of the arrow, and while passing listlessly that way he came upon a string, and was struck by an arrow. There was profuse hemorrhage from the wound. On hearing of this the *shikaree* who had set the arrow soon came to the spot, liberated the arrow from the thigh, and applied some native drug to counteract the poison on the arrow-head. On admission the wound was full of purulent, offensive matter. It was syringed out with sublimate lotion and dressed. In one week the wound looked healthy and was fairly granulating.

But on the 11th—*i. e.*, the twenty-first day after receipt of the wound—the patient had symptoms of lockjaw (trismus). He could not open his mouth to the fullest extent. He was at once put under the influence of bromide of potassium and chloral hydrate. He used to have sound sleep, but there was no diminution of the spasms of the masseters. There were no other symptoms of tetanus. The patient could with slight difficulty swallow liquid or semi-solid food, as rice and milk. There was slight stiffness of the sterno-mastoids. The spasms of the masseters gradually became less troublesome, so that the patient could open his jaws to a greater extent.

On the 21st April the spasm was so troublesome that the patient could not open his jaws to the extent previously done.

On the 24th the patient was laid up with an attack of dysenteric diarrhea, which prostrated him to a dangerous extent. Under appropriate treatment it stopped on the 30th, when the patient passed formed stools.

By the 9th May the patient was strong enough to walk about with the help of a stick and could open his jaws, but there was still present slight contraction of the sterno-mastoid. The wound had by this time completely healed up.

On the 14th the patient had an attack of acute diarrhea. The stools were watery. The diarrhea continued on increasing. The right foot became edematous. And in spite of best treatment and careful nursing the patient's condition became worse day by day.

20th.—The patient was prostrated to an alarming extent. The eyes were sunken, the pulse small and weak. Stimulants had to be pushed on, but it proved of no avail, and the patient died on the 21st.

Is it not a case of tetanus? I should be inclined to answer the question in the affirmative. But we are then to understand that in tetanus the spasms may affect the muscles of the jaws only, and not necessarily all the voluntary muscles, as averred by medical authorities. Spasms of the muscles of the jaws cannot be fatal unless it interferes with free deglutition. In the present case the patient could all along swallow liquid food. The fatal diarrhea was no doubt the result of the action of some poison—maybe the poison on the arrowhead. The question is an intricate one. I leave it to the readers for their opinion.

